# Clinical characteristics and analysis of risk factors for disease progression of COVID-19: A retrospective Cohort Study

**DOI:** 10.7150/ijbs.50654

**Published:** 2021-01-01

**Authors:** Zhengtong Lv, Shubin Lv

**Affiliations:** 1 Beijing Hospital, National Center of Gerontology; Institute of Geriatric Medicine, Chinese Academy of Medical Sciences, P.R. China.; 2 Graduate School of Peking Union Medical College and Chinese Academy of Medical Sciences, P.R. China.; 3 Department of Respiratory Medicine, Tianjin Binhai New Area Dagang Hospital, Tianjin, P.R. China.

**Keywords:** COVID-19, SARS-CoV-2, clinical characteristics, risk factors

## Abstract

**Objective:** Since December 2019, an outbreak of coronavirus disease 2019 (COVID 19) has been experienced from Wuhan, China to the world. A retrospective cohort study was conducted to summarize the clinical characteristics of patients with COVID-19 and to explore the risk factors affecting the disease duration in Jiangan Fangcang shelter hospital, Wuhan, China.

**Methods:** Clinical characteristics of 409 patients with COVID-19 were retrospectively analyzed. We describe the clinical characteristics and distribution of discharge time or transfer time for each patient. Then we performed univariate and multivariate Cox regression analysis to identify potential risk factors for progression from non-severe to severe COVID-19 or death.

**Results:** The median disease duration of all patients was 23 days (IQR 19-28). The main symptoms of the patient were fever (95.6%), cough (74.3%), tiredness (21.5%), and so on. Comorbidities mainly included hypertension (30.6%) diabetes (17.6%) and heart disease (12.5%). The univariate Cox regression analysis showed that old age, number of symptoms, the combination of hypertension, heart disease and pulmonary disease were associated with the progression of disease. The multivariate Cox regression analysis showed that old age (HR: 7.294; 95% CI: 1.442-36.888; *P* = 0.016), the combination of hypertension (HR: 2.230; 95% CI: 1.090-4.562; *P* = 0.028) and heart disease (HR: 2.650; 95% CI: 1.079-6.510; *P* = 0.034) were independent risk factors for progression of COVID-19.

**Conclusions:** The age of the patient, the combination of hypertension and heart disease were independent risk factors for the progression of COVID-19. Cautions should be raised for patients with these risk factors.

## Introduction

In December 2019, an unexplained pneumonia outbreak occurred in Wuhan, Hubei province, China 1. On January 7, Chinese scientists isolated a novel coronavirus from pneumonia patients infected with these viruses, officially called the severe acute respiratory syndrome coronavirus 2 (SARS-CoV-2) 2. Since the WHO emergency committee declared it a global health emergency, more than 200 countries have reported coronavirus disease 2019 (COVID-19).

After COVID-19 infection, the patients were characterized by fever, cough, dyspnea, bilateral lung infiltration, and acute respiratory injury 3-5. Up to now, most studies have focused on the prevention and treatment of severe patients, which may develop into respiratory failure or even death 6. However, according to epidemiological investigation, only 20% of the total number of patients are seriously ill, most of whom are non-severe COVID-19 patients (about 80%) 7. In view of the strong infectivity of SARS-CoV-2, effective treatment of non-severe COVID-19 patients is an important means to prevent the conversion of such patients to severe COVID-19 and even death, which is conducive to reversing the overload of hospitals and preventing the large-scale spread of the epidemic.

To this end, the Chinese government has set up 14 Fangcang shelter hospitals in Wuhan for the treatment of non-severe COVID-19 patients 8. In this study, we retrospectively analyzed the COVID-19 patients in Wuhan Jiangan Fangcang shelter hospital to explored the risk factors that affected the duration of the disease, so as to formulate individualized preventive and therapeutic methods in advance for high-risk patients, take effective preventive measures as soon as possible, and prevent them from turning to severe or death cases.

## Methods

### Study design and participants

This study was a retrospective cohort study, which included COVID-19 patients in zone B and zone C of Wuhan Jiangan Fangcang shelter hospital from February 2020 to March 2020. Wuhan Jiangan Fangcang shelter hospital was managed by workers of Tianjin national medical team. This study was approved by the institutional ethics board of Tianjin national medical team. Written informed consent was waived by the institutional ethics board of the hospital, but oral consent was obtained from all the patients. This study was strictly reported according to STROBE statement.

### Inclusion criteria, exclusion criteria and other criteria

The inclusion criteria were as follows: (1) all patients were confirmed to be COVID-19 by nucleic acid testing; (2) the disease types were non-severe, including mild (with mild clinical symptoms and no pneumonia on imaging) and common (with fever, respiratory tract infection and other symptoms, and pneumonia on imaging); (3) all clinical data required for the study were complete; (4) adults over 18 years old. The exclusion criteria were as follows: (1) patients who have not been confirmed by nucleic acid testing; (2) the disease types were severe at the time of diagnosis; (3) patients with incomplete clinical data. (4) The patient has a mental illness or other condition that affects their insight. In addition, **Table 1** showed the criteria of patient admission, the criteria of transfer and the criteria of discharge of Fangcang shelter hospital.

### Treatment

The general treatment included bed rest, support treatment, symptomatic treatment, sufficient heat, psychological guidance and masks for all patients during hospitalization. After assessment by the responsible doctor, patients with SpO2≤95% would be given nasal catheter oxygen. Antiviral drugs mainly included Abidol and Oseltamivir. In case of suspected bacterial infection, antibiotics including Cefdinir, Moxifloxacin, Levofloxacin and Azithromycin could be used or treatment. Patients could also receive traditional Chinese medicine treatment, such as Qingfei paidutang. If the patient had fever, physical cooling was recommended firstly. If the temperature was more than 38.5 °C, the patient could take medicine to cool down as appropriate (such as Ibuprofen).

### Outcomes

The disease duration of all patients was recorded (The disease duration defined as from the onset of symptoms to the end of discharge). The basic clinical features, comorbidities and CT findings were also recorded. The primary outcome is the risk factors for progression from non-severe to severe COVID-19 or death.

### Data collection

All patients were asked to fill in simple admission records before admission. All clinical characteristics of patients were obtained by collecting these admission records. The discharge date of all patients was recorded every day. By investigating the original records, the patient's disease duration time could be obtained from the time of symptom onset to the time of patient discharge.

### Sample size calculation

In this study, univariate and multivariate Cox regression analysis were performed to determine the independent effect of risk factors for progression from non-severe to severe COVID-19 or death. For sample size estimation, at least 10 samples are generally required as events per variable 9. There were 17 independent variables in this study, so each group needed at least 170 cases, and the total sample size needed at least 340 cases.

### Statistical analysis

Frequency and percentage are used to describe the categorical variables, and the continuous variables were described by mean and standard deviation. Categorical variables were analyzed using Chi-squared test. Continuous variables were analyzed using Student's *t* test. Variables showing a *P* value < 0.05 in univariable analysis were considered as candidates for the multivariate Cox regression model, and a forward stepwise method was used to determine the final multivariable model. SPSS 19.0 was used for the statistical analysis.

## Results

### Clinical characteristics of all patients

**Figure 1:**
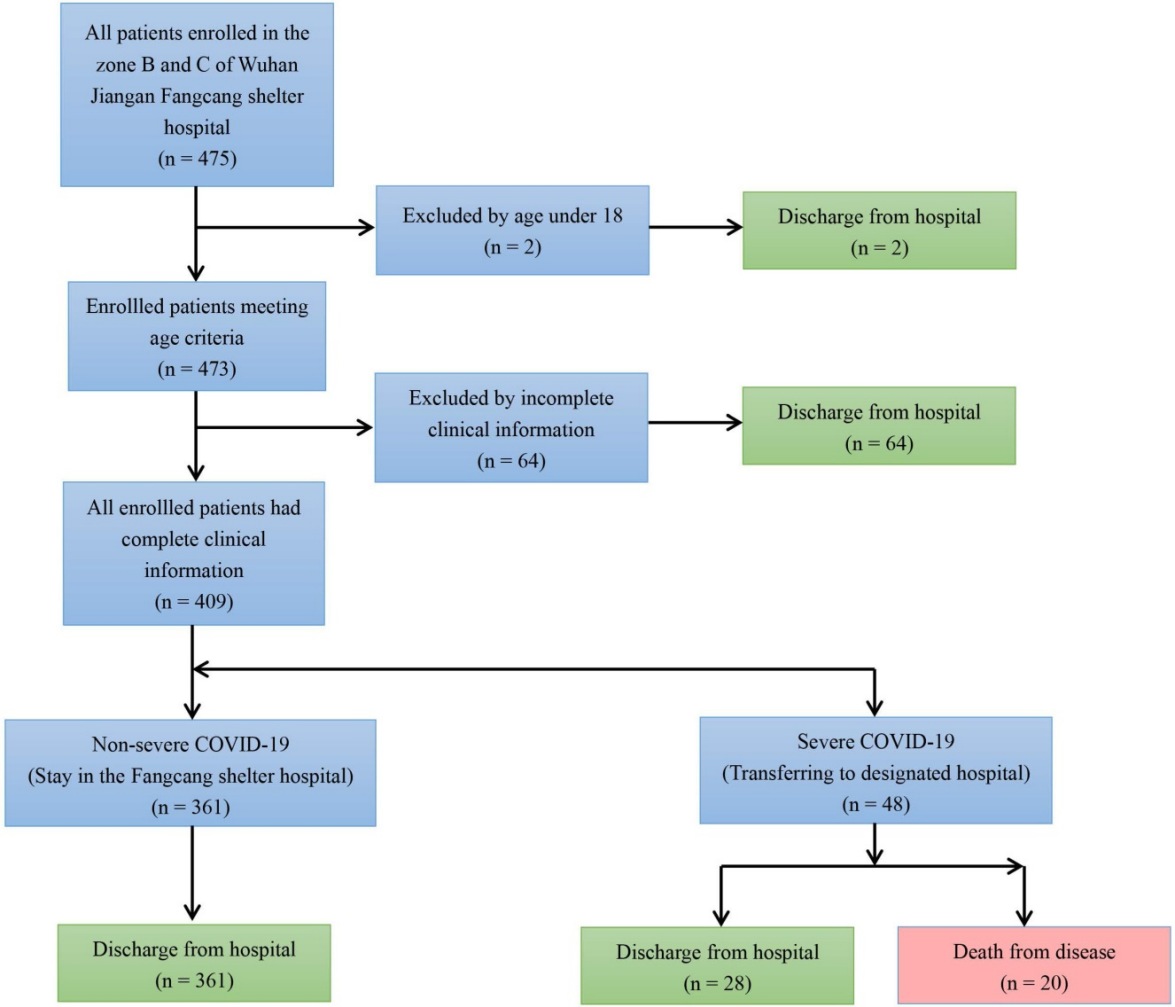
Flow diagram.

**Figure 2:**
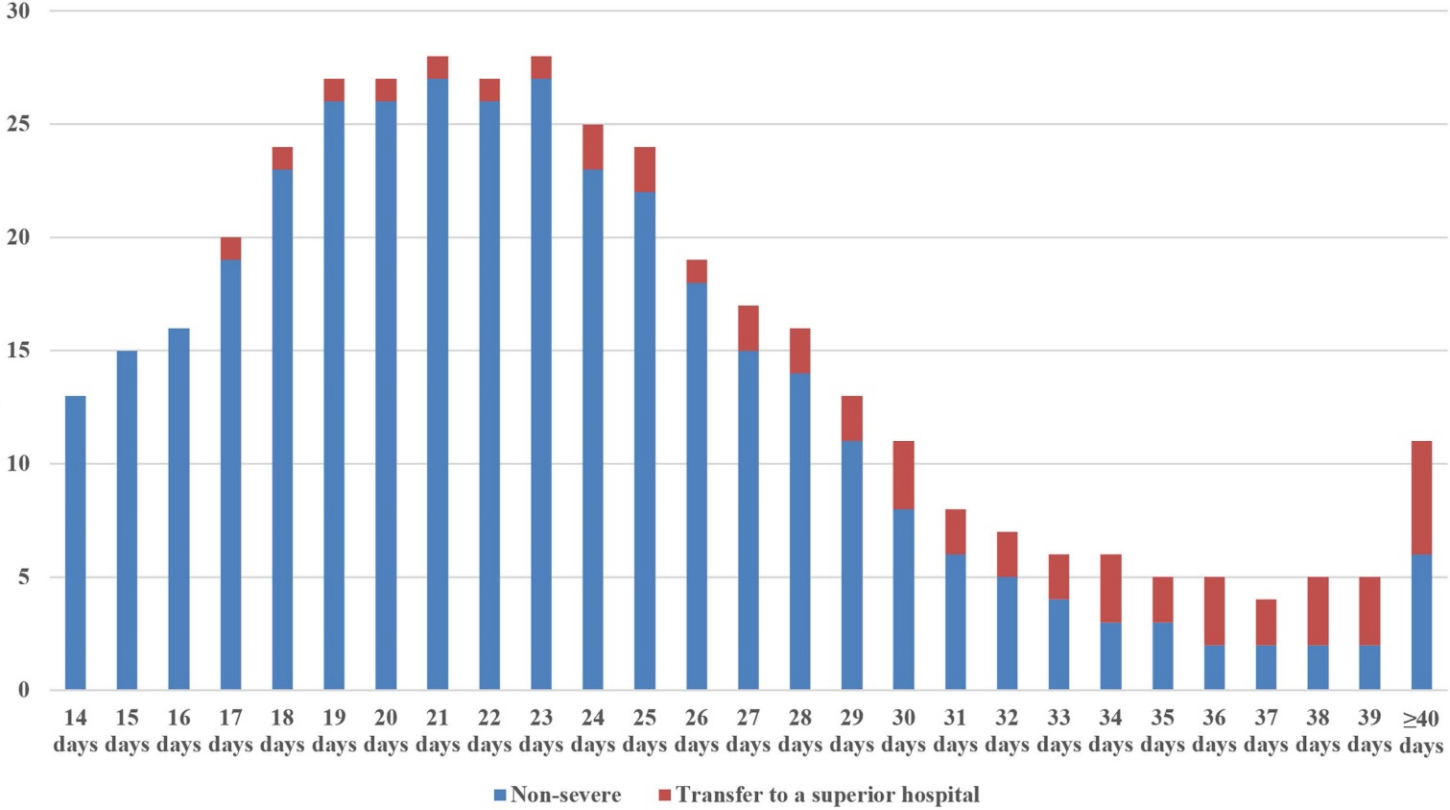
The distribution of discharge time or transfer time for each patient.

By summarizing all admission and discharge records and following strict inclusion and exclusion criteria, a total of 409 COVID-19 patients were enrolled in this study (**Figure 1**). Among them, 48 were severe patients and 361 were non-severe patients. Of the 48 severe patients, 20 died as a result of exacerbations. The rest of the severe patients and all the non-severe patients recovered and were discharged from hospital. **Figure 2** showed the discharge time or transfer time for each patient. The average age of all patients was 50.47 ± 12.43 years. The median duration of all patients was 23 (IQR 19-28) days. The main symptoms of the patient were fever (95.6%) and cough (74.3%), followed by tiredness (21.5%), sputum (18.1%), body aches (15.6%) and diarrhea (7.8%). Comorbidities were present in nearly half of the patients, mainly including hypertension (30.6%) diabetes (17.6%) and heart disease (12.5%). CT signs of pulmonary infection were also present in almost half of the patients, 68.0% of whom showed ground-glass opacity and 60.6% which showed bilateral pulmonary infiltration (**Table 2**).

### The risk factors for non-severe to severe COVID-19 (including death)

Univariate Cox regression analysis indicated that old age (HR: 4.522; 95% CI: 1.067-19.168; P = 0.041), the number of symptoms (HR: 2.055; 95% CI: 1.091-3.871; P = 0.026), the combination of hypertension (HR: 2.544; 95% CI: 1.389-4.659; P = 0.002), heart disease (HR: 2.220; 95% CI: 1.212-4.065; P = 0.010), pulmonary disease (HR: 2.114; 95% CI: 1.070-4.179; P = 0.031) and other comorbidities (HR: 1.838; 95% CI: 1.006-3.360; P = 0.048) were the risk factors for the progression from non-severe to severe COVID-19 (including death) (**Table 3**). Multivariate Cox regression analysis showed that old age (HR: 7.294; 95% CI: 1.442-36.888; P = 0.016), the combination of hypertension (HR: 2.230; 95% CI: 1.090-4.562; P = 0.028) and heart disease (HR: 2.650; 95% CI: 1.079-6.510; P = 0.034) were independent risk factors for progression from non-severe to severe COVID-19 (including death) (**Table 3**).

## Discussion

Wuhan was the epicentre of COVID-19 in China and around the world, in which the confirmed cases accounted for 80% of China's confirmed cases. At the height of the outbreak, the Chinese government quickly set up 16 Fangcang shelter hospitals in Wuhan. Fangcang shelter hospital refers to a novel concept: large, temporary hospitals built by converting public venues, such as stadiums and exhibition centres, into health-care facilities to isolate patients with non-severe novel coronavirus patients 8. Since non-severe patients accounted for 80% of the total number of patients, Fangcang shelter hospitals isolated thousands of patients, provided high-quality medical treatment and care, and fulfilled an important triage function. However, there are also a considerable number of COVID-19 patients under severe or even critical condition complicated with severe pneumonia, acute respiratory distress syndrome, acute respiratory failure or multiple organ failure. It is crucial to identify these high-risk group patients. So, we reported on a single-institution summary about non-severe patients in Wuhan Jiangan Fangcang shelter hospital, with emphasis on the risk factors affecting the disease progression of COVID-19.

Our study showed that old age, hypertension and heart disease were independent risk factors for progression from non-severe to severe COVID-19 (including death). Wang et al. 's study 10 showed that male, elder age, diabetes cardiovascular diseases, chills, dyspnea, SO_2_ value of ≤93%, WBC counts of >10×10^9^/L and large consolidated opacity on CT images were all risk factors for aggravation of illness. Edith Sepulchre et al. 11 found that older age, male gender, comorbidities and dyspnea at admission constituted significantly worse prognosis factors. Feng He et al. 12 found that older age, Wuhan exposure history, diarrhea, chronic kidney disease, elevated myoglobin, elevated white blood cell and C-reactive protein were independent risk factors for severe patients with COVID-19.

A meta-analysis about clinical data of COVID-19 involving 10 epidemiology studies showed that the discharge rate of COVID-19 patients was about 52%, and the fatality rate was about 5% 13. In our Fangcang shelter hospitals, 48 patients were transferred to superior hospitals due to worsening conditions, and 20 of them died in critical condition. The mortality of COVID-19 was 4.89% which was similar to the result of meta-analysis. However, it is remarkable that all of non-severe patients in our and wang et al.'s 10 study was discharged from the Fangcang shelter hospitals, and none died. So, we have enough confidence to cure the non-severe patient. The most difficult task is to treat the severe patient, especially in the absence of evidence supporting an effective drug. Therefore, it is important to identify patients who have the potential to develop severe disease. Early recognition of severe infection may allow early intervention with supportive measures and therapeutics and improve outcomes 14.

First of all, we should pay attention to the age of patients. On the one hand, elderly patients had poor immunity compared with younger patients; on the other hand, elderly patients were more likely to have other comorbidities. Therefore, it was not difficult to understand that old age was one of the factors affecting the course of disease 15. A retrospective study of older COVID-19 patients showed that patients who were 65 years and older, the mortality rate was 34.5% (19/55), which was significantly higher than that of younger patients at 4.7% (7/148). And compared with young patients, older patients had more laboratory abnormalities and comorbidities 16. Therefore, monitoring and supportive treatment of elderly patients should be strengthened.

Second, the number of co-existing symptoms was also correlated with the patients' disease progression and this concept first proposed and discovered in our study. When there were many kinds of symptoms in patients, it meant that the virus load in patients was large, which had seriously affected multiple body systems, not only respiratory system. At this time, the rehabilitation process of the patients needed a long time. With the slow recovery of all systems in the whole body, patients can achieve normal healing, so the course of the patients was prolonged. Therefore, emphasis should be placed on symptomatic and supportive treatment. The relief of objective symptoms was not only beneficial to overcome the disease, but also can enhance patients' confidence, reduce patients' discomfort, anxiety and fear.

Finally, we should pay more attention to the patients with multiple comorbidities, such as hypertension, diabetes, heart disease, pulmonary disease and other comorbidities. Moreover, many studies have shown that patients with these complications were more difficult to treat, more likely to develop disease aggravation, and more likely to lead to death 4,10,16-22. This was similar to MERS-CoV 23. Angiotensin converting enzyme 2 (ACE2) was the gateway to SARS-CoV-1 and SARS-CoV-2 24,25. The association between ACE2 expression and angiocardiopathy was confirmed in previous studies 26-28. This partly explained why hypertension and heart disease may affect the progression of COVID-19. Furthermore, it was well known that high level of renin-angiotensin (RAS) is an important cause of hypertension. In a mouse model, activation of RAS can lead to lung injury 24. It was reasonable to suspect that COVID-19 exacerbated lung injury in patients with hypertension and heart disease by further activating RAS. Diabetes was another risk factor for the prognosis of COVID‐19. Patients with diabetes generally have higher mortality and morbidity to infectious diseases. It may be possibly due to chronic immune system imbalances, metabolic syndrome, or excess nutrition caused by obesity. On the other side, some viruses were diabetogenic themselves 29. A study of SARS found that even non-severe patients who had not received glucocorticoid therapy had higher levels of fasting blood glucose 30. Therefore, one study has suggested that diabetes with SARS-CoV-2 pneumonia may form a vicious circle, which was not conducive to the prognosis of COVID-19 22.

This study has some limitations. First of all, this study was a retrospective cohort study, lacking the validation of prospective studies. Secondly, when we collected data, a large proportion of patients are excluded due to the lack of relevant clinical data, which may lead to potential selection bias. Finally, due to the limitations of the facilities in Fangcang shelter hospital, routine blood and biochemical tests were not performed on all patients during their hospitalization. Therefore, there is a lack of patients' laboratory examinations. According to the results of previous studies, some laboratory indicators may also be risk factors for the prognosis of patients 17,31.

## Conclusion

Patients with older age, hypertension, and heart disease were more likely to deteriorate into severe COVID-19 or even death. These risk factors, as early warning indicators, can alert careful observation and early intervention to prevent disease progression and reduce mortality.

## Figures and Tables

**Table 1:** The criteria of patient admission, the criteria of transfer and the criteria of discharge of Fangcang shelter hospital

No.	Admission criteria	Transfer criteria	Discharge criteria
1	Mild signs or symptoms (mild clinical symptoms, imaging shows no signs of pneumonia) to moderate signs or symptoms (fever, respiratory tract symptoms, imaging shows pneumonia).	Respiratory distress, breathing rate≥30 beats per min	The body temperature is normal for more than 3 days.
2	Have the ability to live and walk independently.	SpO2≤93% in resting state.	Respiratory symptoms improved significantly.
3	No severe chronic diseases, including heart, liver, lung, kidney, brain and other important organ diseases.	PaO2/FiO2 ≤ 300 mmHg(1 mmHg = 0.133 kPa).	Imaging showed significant absorption of lung inflammation.
4	No history of mental illness.	After treatment, the body temperature remained above 38.5 °C for more than 2 days.	Negative test of respiratory virus nucleic acid for 2 consecutive times (sampling interval of at least 1 day).
5	SpO2>93% and breathing rate <30 beats per min in resting state.	Have severe chronic diseases, including heart, liver, lung, kidney, brain and other important organ diseases (also including hemodialysis patients).	Without additional oxygen absorption, SpO2>95%.
6	Other cases need special explanation.	No independent living ability.	The duration of the disease has exceeded 14 days.
7		Suffering from mental illness, mania, etc.	
8		Other cases need special explanation.	
**Note:**	All the above conditions must be met at the same time.	Patients who meet one of the above criteria will be transferred.	All the above conditions must be met at the same time.

**Table 2:** Clinical characteristics of severe and non-severe COVID-19 patients

Items	All patients (n = 409)	Severe patients (n = 48)	Non-severe patients (n = 361)
**Gender**			
Male	188, 46.0%	24, 50.0%	164, 45.4%
Female	221, 54.0%	24, 50.0%	197, 54.6%
**Age**	50.47±12.43	62.98±6.80	48.80±12.05
**Disease type**			
Mild	46, 11.2%	0, 0%	46, 12.7%
Common	315, 77.0%	0, 0%	315, 87.3%
Severe	48, 11.8%	48, 100%	0, 0%
**Fever**			
Yes	391, 95.6%	46, 95.8%	345, 95.6%
No	18, 4.4%	2, 4.2%	16, 4.4%
**Cough**			
Yes	304, 74.3%	38, 79.2%	266, 73.7%
No	105, 25.7%	10, 20.8%	95, 26.3%
**Tiredness**			
Yes	88, 21.5%	11, 22.9%	77, 21.3%
No	321, 78.5%	37, 77.1%	284, 78.7%
**Sputum**			
Yes	74, 18.1%	10, 20.8%	64, 17.7%
No	335, 81.9%	38, 79.2%	297, 82.3%
**Body aches**			
Yes	64, 15.6%	12, 25.0%	52,14.4%
No	345, 84.4%	36, 75.0%	309, 85.6%
**Diarrhea**			
Yes	32, 7.8%	9, 18.8%	23, 6.4%
No	377, 92.2%	39, 81.3%	338, 93.6%
**Number of symptoms**	2 (1-5)	3 (1-4)	2 (1-5)
**Hypertension**			
Yes	125, 30.6%	23, 47.9%	102, 28.3%
No	284, 69.4%	25, 52.1%	259, 71.7%
**Diabetes**			
Yes	72, 17.6%	15, 31.3%	57, 15.8%
No	337, 82.4%	33, 68.8%	304, 84.2%
**Heart disease**			
Yes	51, 12.5%	9, 18.8%	42, 11.6%
No	358, 87.5%	39, 81.3%	319, 88.4%
**Pulmonary disease**			
Yes	17, 4.2%	4, 8.3%	13, 3.6%
No	392, 95.8%	44, 91.7%	348, 96.4%
**Other comorbidities**			
Yes	57, 13.9%	9, 18.8%	48, 13.3%
No	352, 86.1%	39, 81.3%	313, 86.7%
**CT ground-glass opacity**			
Yes	278, 68.0%	39, 81.3%	239, 66.2%
No	131, 32.0%	9, 18.8%	122, 33.8%
**CT bilateral pulmonary infiltration**			
Yes	248, 60.6%	36, 75.0%	212, 58.7%
No	161, 39.4%	12, 25.0%	149, 41.3%
**Disease duration**	23 (19-28)	37 (32-43)	22 (19-26)

**Table 3:** Univariate and multivariate Cox regression survival analysis of risk factor for progression to severe COVID-19

Items	Univariate analysis			Multivariate analysis		
	HR	HR.95% CI	*P*	HR	HR.95% CI	*P*
Age (>50 vs. ≤50)	4.522	1.067-19.168	0.041*	7.294	1.442-36.888	0.016*
Fever (Yes vs. No)	1.391	0.331-5.837	0.652	0.614	0.109-3.488	0.579
Cough (Yes vs. No)	1.251	0.621-2.520	0.531	1.327	0.523-3.366	0.551
Sputum (Yes vs. No)	1.524	0.833-2.790	0.172	1.734	0.778-3.865	0.178
Tiredness (Yes vs. No)	1.125	0.625-2.026	0.694	0.606	0.237-1.551	0.297
Body aches (Yes vs. No)	1.949	0.994-3.822	0.052	2.403	0.962-6.007	0.061
Diarrhea (Yes vs. No)	1.570	0.751-3.283	0.231	1.822	0.716-4.634	0.208
Number of symptoms (>3 vs. ≤3)	2.055	1.091-3.871	0.026*	2.500	0.676-9.241	0.170
Hypertension(Yes vs. No)	2.544	1.389-4.659	0.002*	2.230	1.090-4.562	0.028*
Diabetes (Yes vs. No)	1.291	0.698-2.388	0.416	1.548	0.739-3.240	0.246
Heart disease(Yes vs. No)	2.220	1.212-4.065	0.010*	2.650	1.079-6.510	0.034*
Pulmonary disease(Yes vs. No)	2.114	1.070-4.179	0.031*	0.858	0.327-2.249	0.755
Other comorbidities (Yes vs. No)	1.838	1.006-3.360	0.048*	1.027	0.510-2.069	0.941
CT ground-glass opacity (Yes vs. No)	1.272	0.525-3.084	0.594	0.955	0.367-2.487	0.925
CT bilateral pulmonary infiltration (Yes vs. No)	1.199	0.618-2.327	0.592	1.044	0.495-2.203	0.909

## Abbreviations

COVID 19: coronavirus disease 2019
SARS-CoV-2: severe acute respiratory syndrome coronavirus 2
ACE2: Angiotensin converting enzyme 2
RAS: renin-angiotensin
